# On Diseases of the Liver

**Published:** 1846-02

**Authors:** 


[TO BE CONTINUED.]

---

ART. V.—*On Diseases of the Liver.* By GEORGE BUDD, M.D., F.R.S., Professor of Medicine in King's College, London; and Fellow of Caius Cambridge. *With colored Plates and numerous Woodcuts.*—Philadelphia: Lea & Blanchard. 1846. 12mo. pp. 392.

It is not a great many years since the Liver figured conspicuously in every system of pathology. The influence of this great organ was recognised in almost every disease. We had treatise after treatise upon liver complaints; and fevers, dropsy, dyspepsia, and a host of other maladies were traced to congestion of the portal circle. In derangement of the liver, and cutaneo-hepatic sympathy, and want, or disorder of hepatic secretion, we found an explanation of half the ailments that flesh is heir to.

But "a change came o'er the spirit of our" medical literature; and it is now a long time since we had a volume on the diseases of the Liver. The Lungs, the Heart, the Kidneys, the Skin, and the Nervous System, have usurped its place, and been extorting the attention long bestowed upon it.

This, perhaps, is not difficult to account for. Medicine, unquestionably, is assuming more the character of an exact science. The profession had grown weary of the vague conjectures respecting hepatic diseases, and hepatic sympathies, while it was led away to the affections of the chest, for the investigation of which new and more accurate methods had been discovered; and to those of the skin and kidneys, which were either visible, or afforded, at least, some index to their nature in one of the excretions. The changes wrought by disease in the lungs and heart could be detected by the ear; and some knowledge of the lesions of the kidneys might be gained by the analysis of the urine; but percussion threw very little light on the morbid changes in the liver, and its secretion could not he collected and analysed. Thus, while precise information was attained in respect to the physical condition of other organs, to detect and distinguish the diseases of the liver, practitioners had little more than the signs of functional disturbance. Hence, for so long a time, no addition has been made to the number of our guides in the discrimination and treatment of these disorders. The work before us has appeared opportunely, and will meet with a hearty welcome from the profession.

Very recently, this author remarks, "two of the impediments to the study of diseases of the liver have been in some degree removed." He refers to the precise composition of the bile, with a knowledge of which modern chemistry has furnished us, and to the insight we have gained by the microscope into the intimate structure of the liver. He begins his work with an account of the minute organization of that viscus, some of the points relating to which have only lately been made out. We cannot follow him in this interesting description, but will not leave the subject without reference to one point—the office of the *nucleated cells in effecting secretion*, which is now considered an established fact in physiology. The author remarks on this head:—

"It is not in the liver only that the cells perform this office, for it seems established as a general law, and it is certainly one of the highest and most interesting which the study of minute structure has yet disclosed—that all true secretion, whether in animals or in plants, is effected by the agency of cells; that, 'however complex the structure of the secreting organ, these nucleated cells are its really operative part.' In each secreting organ, the secreting cells have a peculiar power to form, or to withdraw from the blood, the secretion proper to the part.

"In such of the glands of animals as have excreting ducts, the nucleated cells withdraw from the blood the peculiar principles of the secretions, which they elaborate more or less, and then, in one way or other, whether by bursting, or dissolving, or by some unknown mode, discharge them through the excreting duct."

In regard to the quantity of bile secreted by the human subject, we are in possession of no certain information, some physiologists rating it at a very few ounces, while Haller and Burdach have estimated it at from seventeen to twenty-four ounces. There can be no question that the amount varies in different individuals, and in the same person under different circumstances. That the quantity rises in some cases to the estimate of Burdach and Haller, is shown by the following extraordinary case, quoted by the author from the Medico-Chirurgical Transactions:—

"A strong, healthy man, a thatcher, fifty-four years of age, injured himself by lifting a heavy ladder, on the 28th of August, 1843. When seen by Mr. Barlow, the same day, he complained of so much pain in the region of the liver that a rupture of that organ was apprehended. He was very faint, in a cold sweat, and the pulse could scarcely be felt. Some brandy and water was given him, and he recovered sufficiently to be taken to his own house, which was about three miles distant. Five grains of calomel and a grain of opium were given him at night, and an ounce of castor oil the following morning, which operated and produced several natural evacuations.

"On the 29th he was bled, and continued the calomel and opium, with a dose of saline mixture, every five hours.

"On the 30th it was observed that the evacuations from the bowels were white and without bile, while the urine was dark, as in jaundice, Five grains of blue pill were ordered every six hours.

"As the pain in the region of the liver continued, the bleeding was repeated at different times, and a blister was applied over the right hypochondrium. The same medicine was continued till the 15th of September, when a swelling, the size of a walnut, was observed over the region of the liver. This gradually increased, and on the 9th of October, was so large and caused so much pain from distension, that it was thought proper to tap it. Seven quarts of fluid were drawn off, which from its color and taste appeared to be pure bile. The pain was instantly relieved, and the swelling entirely subsided.

"The fluid collected again, and it was necessary to repeat the tapping on the 21st of the same month, when six quarts and a half of fluid were drawn off. This fluid was analyzed by Dr. Pereira, Dr. G. O. Rees, and Mr, Taylor, and found to be composed in great part of bile. Dr. Rees guessed the proportion of bile in the fluid to be at least eight parts in ten.

"On the 31st of October he was tapped again, and seven quarts were drawn off. On the 9th of November the operation was repeated for thə fourth time, when six quarts were withdrawn. On the 18th of November he was taken to St. Bartholomew's Hospital, and tapped again, when nine pints of fluid escaped. The cyst was not emptied as on the former operation, and he suffered extreme pain from the tapping, which he had not previously done. On the following day, bile appeared in his stools, and the urine was lighter colored. On the 3d of December, the motions were of proper color, containing plenty of bile. The swelling gradually subsided, and towards the end of the month he became quite convalescent. In the beginning of February he was able to walk eight or ten miles; and when an account of his case was presented to the Society, appeared to be in good health."

Whatever may be the fate of certain conjectures respecting the final destination of the bile, it is impossible to regard it as purely excrementitious. In the first place, only a very small quantity of it is evacuated by the bowels, and this is conclusive against that supposition. But the fact that it is emptied so high up into the alimentary canal shows that it is designed to take part in the nutritive function. Digestion cannot be completed without it, and one of its purposes, doubtless, is to aid in the perfection of that process; nor is its relation to respiration less direct and fundamental. Whatever embarrasses the lungs affects the liver. If they perform their function less efficiently, it has more to do. A change of climate, which implies a change in reference to the consumption of oxygen, is felt especially by the liver. Imperfect oxidation devolves more labor upon the liver, the office of which is to depurate the blood of various elements, the retention of which would be hurtful to the system. Exercise and diet, in like manner, influence the secretion of this organ, by affording to it more or less carbonaceous matter to be removed in proportion to their amount. Our author gives illustrations of this view of the subject in the following passage:

"Thus, for example, may be explained many of the bilious disorders of hot climates. If, in such climates, the food be not regulated in accordance with the smaller needs of the economy as to animal heat, an excess of bile is formed, and disorder of the stomach and intestines—bilious vomiting, and diarrhœa—are the consequence.

"Hence, also, the general repugnance to rich meats, and the greater tendency which these and spirits unquestionably have to produce disease of the liver, in hot seasons and in tropical climates.

"In the same way may be explained the greater frequency of bilious disorders in middle life, when men begin to take less exercise, and their respiration becomes less active, while on the other hand, the tendency to indulgence at table but too often increases.

"We may also often see inverse evidence of these relations in the effect of pure air and active exercise, in relieving various disorders that result from repletion, and from the retention of principles, which if not burnt in respiration, should pass off by the liver as bile. Every sportsman must have remarked the effect of a single day's hunting in clearing the complexion. It has, no doubt, much the same effect on the liver, as on the skin."

Most persons, our author concludes, "have more liver, as they have more lung, than is absolutely necessary." But in others, this viscus is just competent to the task of purifying the blood from its bilious principles, when in healthful activity. Such persons are born with a tendency to hepatic derangements. Slight causes derange the balance, and they suffer for the want of due bilious secretion, when they change their climate, indulge in gross living, take too little exercise, or become costive. They grow bilious, sallow, and dyspeptic, because the liver is not depurating the blood. Exercise, laxatives, and reduction of diet are followed by relief,—the first promoting a more active combustion of the carbonaceous elements; the last giving the liver less to do. The action of medicines on the liver elicits from our author the following remarks:

"Various medicines seem to fulfil to a certain extent the 2d object, that rendering the liver more active, and increasing in this way the secretion of bile. Mercury, iodine, muriate of ammonia, and taraxacum, have undoubtedly an action of this kind. The first and the last of these medicines, especially, have long been in this country the chief resources of the physician in the treatment of chronic hepatic disorders. The marked temporary benefit often resulting from mercury given for this effect has from the difficulty of distinguishing the various diseases of the liver, and the consequent indiscriminate use of the drug, led to great evils. This medicine was at one time, by English practitioners, given almost indiscriminately, and long persevered in, for disorders of digestion, many of which did not depend on fault of the liver at all, but on local disease of the stomach or intestines, or on faulty assimilation, the result of debility, which the prolonged use of the mercury but too often increased. Of late, these evils have much abated, but still, before the diagnosis is rightly made, mercury is often tried in cancer, and other incurable organic diseases of the liver, in which this and other powerful and lowering remedies can only do harm."

The following are the heads of the subjects embraced in this volume: Congestion of the liver; inflammation of the liver, gall-bladder, and ducts; softening, fatty degeneration, and scrofulous enlargement of the liver; excessive and defective secretion of bile; gall-stones; cancer, and hydatid tumors of the liver; jaundice.

Congestion of the liver, which fills so large a space in many treatises on the affections of that organ, occupies but seven pages in this work. It is divided into *hepatic-venous* congestion, *biliary* congestion, and *portal-venous* congestion. The anatomical characters of congestion of the liver are deep color and increased size, which, generally, are not attended by pain, and only by a sense of weight and fulness, with a slight jaundice tint. Depending upon mechanical obstruction of the circulation, in the heart or lungs, as is generally the case, the remedies for hepatic congestion are to be addressed rather to the primary affection than to the liver. They embrace purgatives, venesection, diuretics, and diet. Where the congestion has its origin in an altered state of the blood itself, as where it accompanies purpura hemorrhagica, the treatment is still less satisfactory. Congestion from fever and ague is the form most successfully treated.

Dr. Budd holds that inflammation ought not to be divided into acute and chronic, but classified according to the cause producing it. He cites inflammation of the knee in illustration, which may be connected with gout, or with gonorrhœa. In the first case it is curable by colchicum; in the last, that article is inoperative. So in other instances we must know the cause, before we can apply the remedy.

Inflammation of the liver he divides into *suppurative*, *gangrenous*, and *adhesive;* to which he adds inflammation of the veins of the liver, and of the gall-bladder and ducts. Abscess of the liver, the result of suppurative inflammation, may originate in either of these causes; namely: 1st. *a blow;* 2dly. *suppurative inflammation of a vein;* or, 3dly. *ulceration of the intestines*, *stomach*, *or gall-bladder*. To one or the other of these causes, he contends, all cases of hepatic abscess may be traced; and whether he has fully established his position or not, he has, at least, rendered it highly probable.

